# Developmental Aspects of SARS-CoV-2, Potential Role of Exosomes and Their Impact on the Human Transcriptome

**DOI:** 10.3390/jdb9040054

**Published:** 2021-11-29

**Authors:** Navneet Dogra, Carmen Ledesma-Feliciano, Rwik Sen

**Affiliations:** 1 Department of Genetics and Genomic Sciences, Icahn School of Medicine at Mount Sinai, New York, NY 10029, USA; 2 Department of Pathology, Molecular and Cell-Based Medicine, Icahn School of Medicine at Mount Sinai, New York, NY 10029, USA; 3 Division of Infectious Diseases, School of Medicine, Anschutz Medical Campus, University of Colorado, Aurora, CO 80045, USA; ledesmacd@gmail.com; 4 Active Motif, Incorporated, Carlsbad, CA 92008, USA

**Keywords:** SARS-CoV-2, COVID-19, placental transmission, development, exosomes, transcriptome, sub-genomic RNA, RNA-sequencing, mRNA vaccine

## Abstract

With over 4.8 million deaths within 2 years, time is of the essence in combating COVID-19. The infection now shows devastating impacts on the younger population, who were not previously predicted to be vulnerable, such as in the older population. COVID-19-related complications have been reported in neonates whose mothers were infected with SARS-CoV-2 during pregnancy, and in children who get infected. Hence, a deeper understanding of the pathophysiology of COVID-19 during various developmental stages and placental transmission is essential. Although a connection has not yet been established between exosomal trafficking and the placental transmission of COVID-19, reports indicate that SARS-CoV-2 components may be trafficked between cells through exosomes. As the infection spreads, the transcriptome of cells is drastically perturbed, e.g., through the severe upregulation of several immune-related genes. Consequently, a major outcome of COVID-19 is an elevated immune response and the detection of viral RNA transcripts in host tissue. In this direction, this review focuses on SARS-CoV-2 virology, its in utero transmission from infected pregnant mothers to fetuses, SARS-CoV-2 and exosomal cellular trafficking, transcriptomic impacts, and RNA-mediated therapeutics against COVID-19. Future research will establish stronger connections between the above processes to develop diagnostic and therapeutic solutions towards COVID-19 and similar viral outbreaks.

## 1. Introduction

Over 5.18 million deaths and 259.5 million confirmed cases have resulted from the ongoing pandemic of coronavirus disease 2019 (COVID-19), which is caused by severe acute respiratory syndrome coronavirus-2 (SARS-CoV-2) [1,2]. The first cases of COVID-19 were reported in December 2019 in Wuhan, Hubei Province, China, with most people who were infected having an association with a seafood and animal wholesale market [3]. The patients presented with a fever, cough, chest discomfort, and pneumonia, requiring hospitalization and placement on ventilators for support, and with some progressing to death [3]. Samples collected from the patients were submitted for isolation in cell culture followed by RT-qPCR and next-generation sequencing (NGS), which revealed the causative agent to be a coronavirus (CoV) [3,4]. Since then, clinical manifestations have expanded to include immune dysregulation, gastrointestinal illness, and long-term post COVID-19 syndromes [5,6,7,8,9]. As of yet, there is no cure or prophylactic treatment to prevent infection, however, multiple vaccines have been produced and disseminated world-wide that reduce rates of severe illness and death [10]. Recent emergence of SARS-CoV-2 variants pose ongoing concerns in relation to vaccine and treatment efficacy [11,12,13,14]. There has also been interest in elucidating the effects of infection on pregnancy and fetal development, however, knowledge gaps remain. Hence, this review compiles information on SARS-CoV-2 virology, in utero transmission from infected pregnant mothers to fetuses, new findings on possible methods of its cellular trafficking through exosomes, and the transcriptomic impacts of SARS-CoV-2 infection, to inform future studies aimed at a deeper understanding of COVID-19, as well as the development of therapeutic solutions against SARS-CoV-2.

## 2. Emergence of SARS-CoV-2 and Relation to Other CoVs

The sequencing of samples from COVID-19 patients revealed SARS-CoV-2 to be a member of the Coronaviridae family, of the genus betacoronavirus, which includes Middle East respiratory syndrome-related coronavirus (MERS-CoV, which was the causative agent in the outbreaks of respiratory disease in the Middle East in 2012 [15]), and the subgenus sarbecovirus, of which SARS-CoV (associated with the 2002–2003 pandemic and first identified in the Guangdong Province, China [16,17]) is also a member [3,18]. Although SARS-CoV-2 and SARS-CoV are both members of the sarbecovirus genus, SARS-CoV-2 was found to be more closely related to other bat SARS-like betacoronaviruses [3,18,19]. Due to this genetic similarity, bats have been proposed as the reservoir that the virus may have originated from; other animals, including pangolins [20], have been theorized to be potential intermediate hosts, due to genomic sequence similarity [18,19].

### 2.1. General Description of Genes and Proteins

As a member of the betacoronavirus genus, SARS-CoV-2 is an enveloped, positive-sense, single-strand RNA virus. Its genome is approximately 30 kb in length and consists of open reading frames (ORFs) that are commonly shared with other CoVs; these ORFs have the function of coding for accessory, non-structural proteins (nsps). These are arranged in a typical betacoronavirus organization of a 5′ UTR, the replicase complex, a spike (S), an envelope (E), a membrane (M), and nucleocapsid (N) genes which are interspersed with 16 non-structural accessory genes, and a 3′ UTR [3,18,19,21,22]. While the S, E, M, and N proteins have structural functions, the nsps have roles in transcription, translation, RNA processing and proofreading, as well as host immune evasion and response modulation; some of these nsps, however, have unknown functions [21].

### 2.2. Replication Cycle from Entry to Egress

The CoV replication cycle begins with the S protein binding and engaging the host cell entry receptor and initiating the membrane fusion process, a process which also determines host and cell tropism, as well as specific targeting, and pathogenicity [21]. In the case of SARS-CoV-2, the receptor is the angiotensin-converting enzyme 2 (ACE2), which is also the entry receptor that is used by other human CoVs, including SARS-CoV [18,19,21]. The S protein is functionally divided into S1 and S2, with the S1 domain located on the spike surface and containing the receptor-binding domain (RBD), which engages the ACE2 receptor. Following this binding, the transmembrane S2 domain undergoes conformational rearrangements to mediate the fusion of the viral and host cell membranes [21]. The proteolytic cleavage of S proteins by a host protease, called furin, and activation by type II transmembrane serine protease (TMPRSS2), located on the host cell surface, are required for these conformational changes that lead to fusion and entry into the host cell cytoplasm [21,23,24].

Following fusion and uncoating, the genomic RNA is released into the cytoplasm of the host cell, where ORF1a and ORF1b are translated into polyproteins pp1a and pp1b, which undergo proteolytic cleavage to yield nsp1-16 by proteases that are located within nsp3 and nsp5 [21]. Nsp1 targets the translational machinery, while nsp2-16 assemble to form the replication and transcription complex (RTC), and nsp1-11 take part in RTC supportive functions, including modulating the host immune response and intracellular membranes, as well as serving as co-factors for replication. Nsp12-16 are also involved in RNA translational functions. Nsp12 is the RNA-dependent RNA polymerase (RdRP), which along with nsp7-8, perform RNA synthesis. Nsp14 is involved in RNA proofreading functions by use of its 3′-5′ exonuclease activity. Nsp10, 13-14, and 16 are involved in capping functions [21].

Full-length negative sense RNA copies are synthesized, which serve as templates to produce positive sense genomic RNA, which are used to translate more nsps or RTC, or are packaged into virions. Negative sense subgenomic RNA (sgRNA) and positive sense mRNA (sg mRNA) are also produced, which lead to the translation of structural S, M, N, and accessory proteins. Replication compartments that are composed of double membrane vesicles (DMVs) are formed during the replication cycle, which are also called replication organelles (Ros) [21]. These structures are a likely site of RNA synthesis, providing a mostly enclosed space where RNA synthesis can take place. The presence of double stranded RNA (dsRNA) intermediates within these compartments and the discovery of DMV pores from which dsRNA intermediates could move out of the organelles and into the cytoplasm support this theory [25,26,27]. In preparation for egress, structural protein assembly has been suggested to take place in the endoplasmic reticulum (ER)-to-Golgi intermediate compartment (ERGIC) which then progresses to exocytosis [21]. However, recent findings suggest an alternate route of egress by use of lysosomal trafficking [28] and exosomes, which is described later in this review.

## 3. SARS-CoV-2 Infection and Immunity in Pregnancy and Fetal Development

### 3.1. Impact of COVID-19 on Fetal Development and Children

The transmission of COVID-19 from infected pregnant mothers to fetuses has been reported, although at a very low frequency. Pediatric cases of COVID-19 with devastating outcomes have been reported [29,30,31,32,33,34]. “Long COVID”, one of the severe outcomes of infection, in which symptoms persist for 5 weeks or longer following an acute SARS-CoV-2 infection, has been reported in children similarly to adults [33]. Children have also presented with pediatric inflammatory multisystem syndrome that was temporally associated with COVID-19 (PIMS-TS) [33]. It is of grave concern to observe that the nasopharynx of children less than 5 years old with mild to moderate COVID-19 contain more SARS-CoV-2 viral RNA when compared to older children and adults [35], which may impact its transmission [36].

SARS-CoV-2 infection induces fetal distress, as well as substantial morbidity and mortality in children [37,38]. Currently, sufficient data is not available to confirm adverse effects upon future generations derived from individuals who were positive for COVID-19 during pregnancy. However, the observations of multisystem inflammatory syndrome in children (MIS-C) [30] and other associated complications warrant further research to understand the complete range of effects of COVID-19 in children, in utero development, and on SARS-CoV-2 cellular trafficking mediated by exosomes during in utero and perinatal developmental stages.

Maternal to fetal transmission is one of the various devastating effects of COVID-19 and a major area of concern which has not been fully explored. Although limited reports exist on the in utero transmission of COVID-19, those studies indicate a diverse spectrum of disease outcomes. However, the short-term and long-term impacts on neonates whose mothers were affected with COVID-19 during pregnancy is not well understood [39] in the absence of large volumes of data. Recent studies, however, show that neonates are adversely affected [40]. In this direction, Mullins et al. presented a review on pregnant women who were infected by SARS-CoV-2 during pregnancy and the incidence of fetal distress [41]. The review discusses 32 women who were affected by COVID-19 during pregnancy, where 22% of newborns delivered were asymptomatic, while 6% underwent admission to the intensive care unit (ICU), including one who was subjected to extracorporeal membrane oxygenation [41]. Interestingly, no vertical transmission was reported in the neonates in this report [41].

Another review of the literature, on 564 pregnant women who were infected with COVID-19, showed that 18 of the 549 neonates who were tested were positive for infection [42]. Interestingly, another review on 336 neonates who were screened for COVID-19 showed that only 15 tested positive, however, that only one of their concomitant amniotic fluid samples tested positive [43]. Likewise, another study supports the possibility of SARS-CoV-2 vertical transmission, although this is of low likelihood [44]. The study tested 31 mothers with COVID-19 and their newborns, and detected the SARS-CoV-2 genome in one umbilical cord blood sample, two at-term placentas, one vaginal mucosa, and one milk sample [44]. Interestingly, they further found specific anti-SARS-CoV-2 IgM and IgG antibodies in one sample of umbilical cord blood and milk, and three documented cases of vertical transmission with SARS-CoV-2 infection, as well as a strong inflammatory response [44]. A cross-sectional study on 63 pregnant women with mild to moderate COVID-19 at a single hospital showed that two vaginal secretion samples and one placental sample tested positive [45]. Within one day of birth, two neonates tested positive for COVID-19, and IgG and IgM, but RT-PCR test was negative [45].

To further study pregnancy outcomes, the transmission of COVID-19 from pregnant mothers has been investigated by several other groups as well [46,47,48,49,50,51,52,53,54,55,56]. A pregnant individual with SARS-CoV-2 infection reported placental abnormalities that were consistent with severe vascular malperfusion, or loss of blood supply, due to obstruction and pulmonary inflammation of the fetus, which did not survive [53]. This study indicates that the monitoring of coagulation and the inflammatory response in high-risk COVID-19 positive pregnant women may improve outcomes [53].

**Figure 1:**
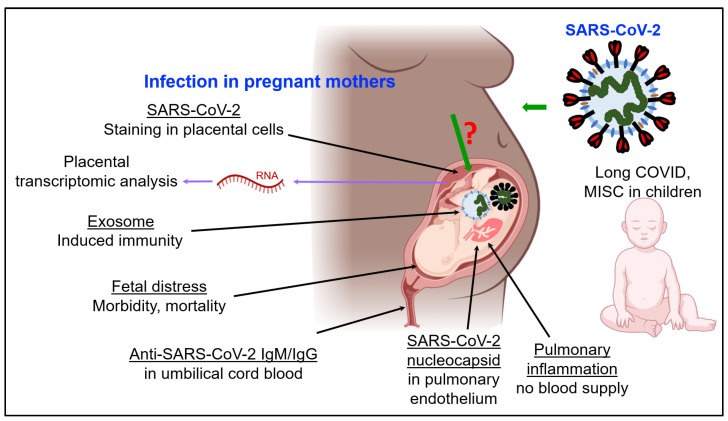
Reported impacts of SARS-CoV-2 infection on fetuses through pregnant mothers, and on children. Abbreviations: MISC multisystem inflammatory syndrome in children. “?” indicates that transmission mechanism is not clearly understood.

A recent study showed SARS-CoV-2 staining in the placental cellular layers during embryonic development [57]. Positive staining for SARS-CoV-2 was detected in the epithelial covering of the placental villi called the syncytiotrophoblast, as well as in the inner cellular layer that gives rise to it, called the cytotrophoblast [57]. This study is the first to report the details of the participation of the cytotrophoblast in the SARS-CoV-2 infection process, which adds to the list of fetal cell types from the placentas of infected mothers where viral staining is detected [57]. Another study has reported the positive immunohistochemistry of a SARS-CoV-2 nucleocapsid within fetal pulmonary endothelium, which indicates vertical transmission [58]. Overall, the above studies demonstrate the current evidence of transmission of SARS-CoV-2 from pregnant mothers to fetuses (Figure 1). In this direction, an important process is cell-to-cell communication via exosomes, which shows evidence of trafficking of viral components between cells; this is discussed below.

### 3.2. SARS-CoV-2 and Fetal Development: The Role of Exosomes

Although it is clear that SARS-CoV-2 infection induces an immune response in pregnant women, the alterations in the fetal immune responses remain a matter of intense debate. In a recent study, 205 infants born to COVID-19-positive mothers were investigated [59]. While only ~10% infants were found to be positive for COVID-19, most studied infant cases had developed immunoglobulin G and M (IgG, IgM) antibodies against SARS-CoV-2 [59]. In another study, no viral RNA was detected in the placentas of COVID-19 positive pregnant women [60]. Furthermore, there seems to be no confirmed cases of intrauterine infection of SARS-CoV-2 from mothers to their fetuses. Although severe illness has been seen in infants younger than 1 year, such cases have had confirmed underlying comorbidities [61]. These findings suggest that vertical infection is rare, and a natural passive immunity is developed in infants who are born to mothers with COVID-19 [59,60].

Exosomes are secreted by all cell types studied to date [62,63,64]. With respect to the placental lineage, exosomes have been investigated from mesenchymal, endothelial, and trophoblastic lineages and have been demonstrated to suppress T-cell expression [65]. Here, we investigated the role of exosome trafficking in utero and their significance with respect to SARS-CoV-2 infections and the subsequent development of an immune response in infants. Exosomes are extracellular nanovesicles (~50–200 nm) of endocytic origin that package cellular constituents; this is likely to maintain cellular homeostasis, but the reason behind their production is unknown [66]. There seem to be two potential hypotheses of exosomal contribution in utero, as well as in fetal development: (1) SARS-CoV-2 infections via exosomes, or (2) in utero development of immunity. While exosomes have been found to carry viral RNA, there seems to be little to no viral replication in utero [60]. This observation discards the first hypothesis that exosomes may induce viral infection in utero.

Alternatively, our second hypothesis regarding exosomes’ role in the development of an immune response is of major interest, and may have numerous implications in utero and in fetal development [67,68]. Exosomes predominantly carry major histocompatibility complex (MHC) class I and II molecules on their surface, which can activate T lymphocytes, and trigger an adaptive immune response [67]. As the placenta promotes the production of exosomes that are enriched in developmental and immune response cargo, the presence of a complete antigen-presentation molecular machinery within exosomes has direct implications on the development of a fetal immune response to SARS-CoV-2 infection [65]. Rising debate surrounding whether exosomes are capable of either a direct or indirect activation of immune response may be correlated to the cell of origin of the given exosomes.

Regardless, accumulating evidence suggests that MHC-I and II molecules on the surface of exosomes can functionally form a complex with antigenic peptides to induce immune activation. The presence of these MHC-I and II molecules may suggest and provide the basis of the important roles of exosomes in the immune cascade. Based on these observations, we reason that the mechanism of MHC secretion through exosomes and their potential role in cell-to-cell communication, targeted function, and immune regulation may target in utero immune development. Finally, we hypothesized the potential use of MHC-exosomes as the extracellular particles of choice, which may lead to therapeutic procedures for immunity development in mothers and infants.

### 3.3. Development and Functions of Exosomes: Biogenesis and Biology

Exosomes are a subclass of extracellular vesicle (Evs) that are released by all cell types and are involved in extracellular communication. Unlike other Evs, exosomes are formed by the inward budding of the membrane of late endosomes, otherwise known as multivesicular bodies (MVBs) [69,70,71]. Subsequently, these MVBs fuse with the plasma membrane (PM) resulting in the release of exosomes to the extracellular environment [72,73]. Given their unique intracellular trafficking pathway, exosomes encapsulate different cargo content [74,75,76,77]. The endosomal sorting complexes required for transport (ESCRT) proteins, along with the Rab (Ras-associated binding) small GTPase family, serve a crucial role in the modulation of exosomal secretion and trafficking [78].

This mechanism starts with the ESCRT-0 protein utilizing hepatocyte growth factor-regulated tyrosine kinase substrate (HRS) to identify and cluster ubiquitinated transmembrane proteins in the endosomal membrane. Once properly localized, the HRS recruits ESCRT-I/II complexes, along with associated proteins (for instance, TSG101, ALIX, VPS4, etc.), for the initiation of MVB biogenesis via budding. Finally, the actual process, involving vesicle scission, is primarily driven by the ESCRT-III protein. Free ESCRT components and ubiquitin molecules are recycled for repeating the process post-scission of the MVBs [74,78].

Following the formation of MVBs, the remainder of the trafficking pathways (comprising the cytoskeleton, molecular motors, and vesicle fusion machinery) are mostly regulated by the Rab family of small GTPases [79]. In particular, both RAB27A and RAB27B are associated with the promotion of MVB docking and fusing to the PM, as well as the vesicle transfer from the Golgi apparatus to MVBs. Likewise, mechanisms involving RAB small GTPases often recruit SNAP receptors (SNAREs), a superfamily of proteins, for the mediation of vesicle trafficking within cells [79,80].

Despite the critical role of the ESCRT complexes, further evidence has demonstrated an alternative, ESCRT-independent, pathway of exosomal packaging and formation [80]. In addition to proteins that are actively involved in exosomal biogenesis (i.e., TSG101, ALIX, RAB proteins, and annexins), other frequently observed exosomal proteins include membrane transport proteins, metabolic enzymes, fusogenic proteins, tetraspanins, heat shock proteins, cytoskeletal proteins (actin and tubulin), lipoproteins, and enzymes (phospholipases).

Nevertheless, exosomes are not the only extracellular vesicles that are released; others which are released are often called apoptotic, micro-, and onco-vesicles [69,71]. All extracellular vesicle cargo leave molecular footprints from their cell of origin; exosomes selectively package proteins and nucleic acids and appear to avoid cellular debris [69,81]. Recent proteomic studies have revealed a set of endocytic, cytoplasmic, and endosomal proteins in exosomes. In contrast, PM-derived vesicles predominantly contain nuclear DNA, mitochondrial DNA, rRNA, and PM-associated proteins [69,81].

### 3.4. Genomic, Transcriptomic, Proteomic, and Lipidomic Landscape of Exosomes

Despite the wide therapeutical and diagnostic applications that have been confirmed today, exosomes were thought to only pertain to cargo cell debris and waste in the early stages of exosomal research in the 1980s [78,82]. Nevertheless, beginning in the 1990s, studies showed results suggesting exosomes’ pivotal role in cell-to-cell communication and as triggers for cancer immune responses [83,84]. The proteomic composition of exosomes is a consequence of their cell of origin and their endosomal molecular pathways. Exosomes are composed of highly enriched tetraspanins (CD9, CD81, CD63) and other endosome-associated proteins (RAB, SNARE, TSG101, ALIX, and ESCRT) [64,70,71]. Much of the proteomic landscape of exosomes has been discussed above in the exosome’s biogenesis section.

Major breakthroughs were marked in 2000′s, as mRNAs and microRNAs were unveiled in exosomes along with their influence on cellular behaviors and functions [81]. In particular, a wide variety of genetic material were gradually identified, including mRNA, ncRNA, miRNA, lncRNA, ssDNA, dsDNA, mitochondrial DNA, and oncogene amplifications [81,85,86,87,88]. In chronic lymphocytic leukemia, exosomes shuttle proteins, lipids, miRNAs and mRNAs to recipient cells, and regulate their transcriptomes and behaviors [89]. Those exosomes are enriched for miR-202-3p, which likely impacts Hedgehog signaling [89]. Exosomes contain mRNA and a variety of non-coding RNA whose alterations are partly reflected in the cellular transcriptome, which indicates the potential of the exosomal transcriptome as a biomarker [90,91]. Exosomes that are derived from the placenta regulate maternal immune tolerance during pregnancy [92]. The transcriptomes and proteomes of exosomes that are derived from avian serum have provided important insights regarding antiviral vaccination [93]. Hence, exosomal transcriptome analysis has the potential to inform about some of the complexities that have been reported in pregnant women who have received COVID-19 vaccines [94]. Exosomes are also significant for other diseases, because their participation in signaling pathways and cell-to-cell communication impacts the tumor microenvironment [62,63,91,95].

Aside from genetic materials, exosomes have also been confirmed to deliver lipids and proteins [74,76]. The inclusion of proteomic components and genetic materials suggests that exosomes have the capability of regulating and triggering specified signaling cascades, thereby altering the transcriptional landscape of the targeted cell. These characteristics enable exosomes to regulate cellular crosstalk and vesicle trafficking for impacting disease progression, the tumor microenvironment, metastasis, and other processes [76].

When compared with their cell or origin, exosomes tend to be enriched in proteins that are located in lipid rafts, including glycosylphosphatidylinositol-anchored proteins and flotillin. Exosomes are enriched in cholesterol, sphingomyelin, gangliosides, ceramides, and phosphatidylserine (PS) [64]. PS and phosphatidylethanolamine (PE) are enriched on the outer membrane of exosomes, whereas PS and PE tend to be depleted in the outer cell membrane. The underlying reason behind the differential lipid composition of exosomes could be credited, in part, to a different membrane curvature from their cell of origin. The large curvature of cells (~2 micron) versus the small curvature of exosomes (~100 nm) may recruit different lipids for their formation.

## 4. SARS-CoV-2 Infection and Exosomal Pathway

### 4.1. Does SARS-CoV-2 Hijack the Exosomal Pathway for Cellular Entry and Exit?

**Figure 2:**
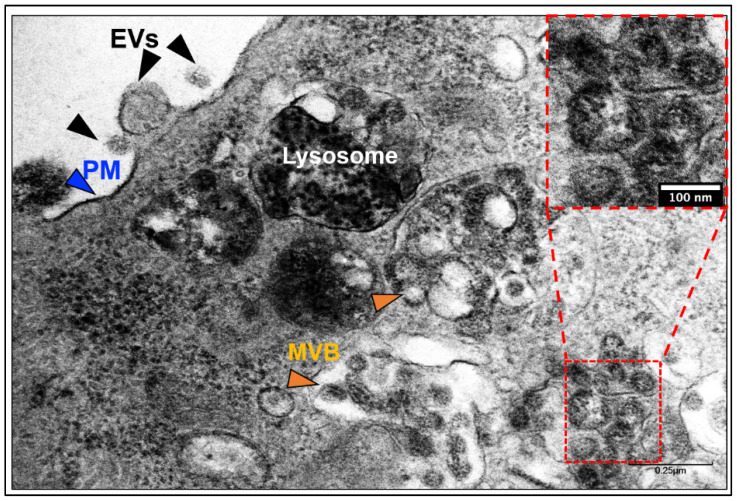
Transmission electron microscopy (TEM) of SARS-CoV-2-infected Vero E6 cells. Exosomes and/or a SARS-CoV-2-like particle can be seen (inset) inside an MVB. Evs—extracellular vesicles, PM—plasma membrane, MVB—multivesicular bodies.

At a first glance under a transmission electron microscope (TEM), both exosomes and SARS-CoV-2 virions appear to be seemingly identical particles (Figure 2 shows SARS-CoV-2 infected Vero E6 cells) [91]. Both exosome and virion are spherical, membrane-enclosed, RNA-packed, ~100 nm size particles. However, when analyzed at the molecular level, both virions and exosomes have unambiguous differences, mainly due to their surface proteins and nucleic acids [96,97]. Nevertheless, whether SARS-CoV-2 mimics the exosomal pathways for entry and secretion is unclear. Addressing this gap in the knowledge will help us better understand the biological pathways of SARS-CoV-2 entry, cellular release of the virion, and novel vaccination strategies.

### 4.2. Entry of SARS-CoV-2 and Exosomes in Cells

Most cells internalize ligands through multiple mechanisms, such as phagocytosis or pinocytosis [98,99]. Additionally, membranous particles can also fuse with the cellular PM, however, this mechanism is less profound in the internalization of influenza viruses [98]. In fact, most viruses are believed to follow the endosomal pathways and avoid membrane-membrane fusion with the cells [98]. This may be because membrane fusion can assimilate viral proteins and lipids with the PM of the cells and leave traces at the cell surface. These leftover traces may help cells to recognize the incoming intruders and be better prepared for the next similar viral invasion.

With respect to the SARS-CoV-2 virus, it is now established that CoVs use the ACE2 receptor for binding with the host cells; gastrointestinal, kidney, and heart tissues express the highest amount of ACE2 [2]. Serine protease TMPRSS2 acts as the priming agent for the S protein [2,96]. It has also been confirmed that the spike protein facilitates viral entry into the host cell [96,97]. Taken together, the most likely mechanism of SARS-CoV-2 entry seems to be a receptor-mediated endocytosis or pinocytosis. Whether the entry is clathrin-dependent or independent remains to studied.

### 4.3. Exit of SARS-CoV-2 and Exosomes from Cells

Recent studies have shown that CoVs use lysosomal, instead of biosynthetic pathways, to exit the cells [28]. Ideally, a late endosome (500–1000 nm) encapsulates the CoV in the cytoplasm of the hijacked cells. Subsequently, the endosome fuses with the PM to release the virus particles to the extracellular environment. This mechanism is identical to the biogenesis and release of exosomes [66,69].

**Figure 3:**
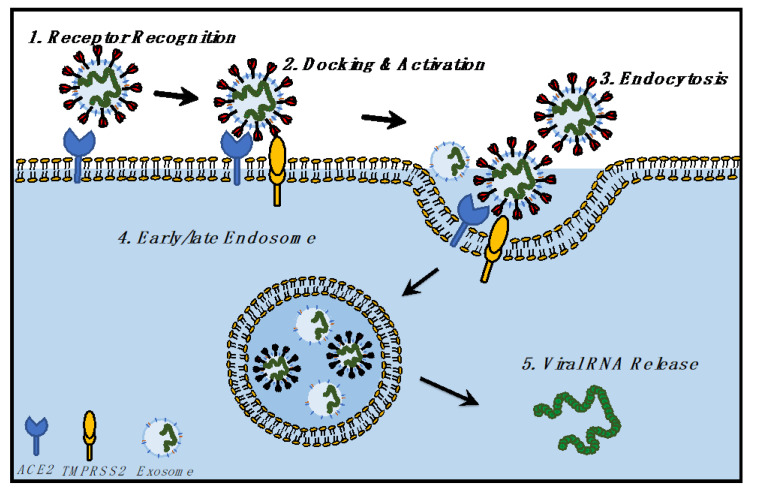
Hypothesis of the biological relationship between SARS-CoV-2 and exosomes, focusing on the identical mechanism followed by SARS-CoV-2 and exosomes for cellular entry and exit.

Here we propose a hypothesis (Figure 3): viruses hijack the evolutionary exosomal pathways, and current vaccination strategies could learn from exosomal intake and release for a better understanding of viral entry, intake, and release. Studies on cancer have shown that exosomes and exosomal RNA impact cellular development, function, and gene expression [100,101,102,103]. Hence, the next section describes the impact of SARS-CoV-2 infection upon the host transcriptome, readouts from host transcriptomic perturbations, and lessons from the viral transcriptome which have been obtained from infected samples of COVID-19 patients.

## 5. RNA-Based Analysis of COVID-19 Patients

Transcriptomic analyses of COVID-19 patients have provided significant insights into the development of various cells of the immune system, COVID-19 pathogenesis, and host responses to SARS-CoV-2 infection. In this direction, this section describes studies involving the RNA-sequencing of tissue samples from COVID-19 patients, and also presents the importance of RNA in therapeutic developments against COVID-19. Studies have reported that: (1) transcriptomic profiles of placental cells show changed expression patterns of *ACE2*, *TMPRSS2*, and *furin* under hypoxic conditions [104], a minimal expression of genes which mediate canonical host cell-entry of SARS-CoV-2 [105]; (2) distinct transcriptional profiles of the host are associated with COVID-19 pathogenesis [106]; (3) transcriptional signatures of the T-cells of COVID-19 patients impact COVID-19 phenotypes [107]; (4) tissue-specific transcriptomes of COVID-19 patients reveal the host response to infection [108]. Transcriptomic studies on African green monkeys (*Chlorocebus aethiops*) show a variation in the immune cell populations of the lung throughout the course of SARS-CoV-2 infection [109]. In addition to the above studies on the host transcriptome, studies focused on viral RNA have shown the presence of SARS-CoV-2 transcripts in at least 8 categories of immune cells [110]. Together, RNA-based studies continue to provide information that is crucial to our understanding of COVID-19, as elaborated below.

### 5.1. Transcriptomic Analysis of Placental Cells in the Context of COVID-19

Transcripts of essential host genes for SARS-COV-2 infection, such as ACE2, TMPRSS2, and furin, were analyzed in various placental cells such as differentiated syncytiotrophoblasts and progenitor cytotrophoblasts. Primary human trophoblasts were cultured under hypoxia or in the presence of dimethyl sulfoxide (DMSO), which are two conditions that impede the in vitro differentiation of cytotrophoblasts to syncytiotrophoblasts [104]. ACE2 and furin expression moderately decreased in both conditions, with these reductions being statistically significant, and the decrease was seen in cytotrophoblasts when compared to differentiated syncytiotrophoblasts [104]. However, under the same conditions, TMPRSS2 expression was elevated [104]. Another study employed single-cell RNA sequencing to study the expressions of ACE2 and TMPRSS2 during pregnancy, by analyzing the placenta and third-trimester chorioamniotic membranes [105]. They reported a minimal expression of the above genes in the placenta [105]. A study found that the expression of placental ACE2, was higher in females with severe COVID-19, however, TMPRSS2 or furin expression were not increased, when RNA was analyzed from placental biopsies of COVID-19-positive females in late pregnancy [51]. Other studies have also focused on an RNA-based analysis of the placenta to understand COVID-19 [111,112].

### 5.2. Transcriptome of Children Affected with COVID-19

A study using RNA sequencing on 12 children and 27 adults with SARS-CoV-2 showed that children have a higher expression of genes for interferon (IFN) signaling, the NLRP3 inflammasome, other innate immunity pathways, and genes associated with immune cells [113]. Pediatric nasal fluids show higher levels of IFN-α2, IFN-γ, IP-10, IL-8, and IL-1β proteins [113]. Single-cell RNA sequencing of COVID-19 patients aged between 4 weeks to 77 years, where 24 were pediatric individuals and 44 were adults, revealed stark differences in the transcriptomes between the two populations [114]. SARS-CoV-2 infection resulted in a phenotype of neutrophil activation, and the expression of proinflammatory genes was greater than in adults, but the proportion of immune and epithelial cells were almost stable in children [114]. Another study using single-cell RNA sequencing on pediatric and adult lung tissues has also reported a unique immune profile in pediatric cases of COVID-19, primarily because their immune system is undergoing development [115]. Overall, the view that bulk or single-cell transcriptomic studies on children affected with COVID-19 reveal significant insights and need to be performed further is collectively shared [116].

### 5.3. Host Transcriptional Profiles and COVID-19 Pathogenesis

The study by Daamen et al. performed a transcriptomic analysis of peripheral blood mononuclear cells, postmortem lung tissue, and bronchoalveolar lavage (BAL) fluid of COVID-19 patients [106]. They found 4245 differentially expressed genes (DEGs) in blood, among which 2166 were upregulated and 2079 were downregulated. The lung transcriptome revealed 2220 DEGs, with 684 upregulated genes and 1536 downregulated genes. Transcriptome of the airway BAL showed 8952 DEGs, where 4052 genes were upregulated and 4900 were downregulated [106].

Gene set variation analysis of the above results revealed an upregulation of the innate immune response pathways in all three of the above components, while downregulation of adaptive immune signatures was observed in the blood and airway BAL only [106]. The lung transcriptome revealed an elevated expression of Type I IFN genes, and an enrichment of Type I and Type II IFN gene signature. The airway transcriptome showed a reduced expression of mitochondrial antiviral-signaling protein, which is a signaling adaptor for RNA virus sensors and is likely involved in SARS-CoV-2-associated viral immune evasion of the host [106]. Several pro-inflammatory chemokines were elevated in the transcriptomes of the blood, lung, and airway. Transcriptomic analysis further revealed that SARS-CoV-2 infection modifies resident tissue populations, because non-hematopoietic cells in the BAL fluid likely reflect viral-induced damage [106]. Overall, the transcriptomic signatures in the blood, lung, and airways revealed significant insights into how the host immune system behaves during the pathogenesis of COVID-19.

### 5.4. Host Transcriptomic Profiles and T Cell Behavior

Single-cell transcriptomic analyses (TA) were performed on respiratory material from COVID-19 patients, which indicated differential CD4 T cell transcriptomics that were likely associated with the sensing of cytokines, triggered by T-cell receptors [107]. Further transcriptomic analysis of CD4 and CD8 T cells supported transcriptional modulation in peripheral blood mononuclear cells, which is induced by antigens. This study was also successful in employing stimulation-induced IFNγ elevation as a surrogate marker to screen for antigen-reactive clonotypes and to functionally validate T-cell receptors which are reactive to SARS-CoV-2 [107].

Hence, the study identified antigen-reactive clonotypes which helped in a process called reverse phenotyping, or “looking back” at phenotypes without the re-stimulation that may functionally change clonotypes. Reverse phenotyping was performed using the T-cell receptor sequence of peripheral blood T cells as natural barcodes, and this process identified phenotypic biases induced by the in vitro antigen re-stimulation of the cells [107]. In simpler terms, reverse phenotyping enabled the authors to obtain a functional readout of antigen-reactive clonotypes, followed by a detection of the supposed phenotype of the cells in the absence of stimulation. Hence, the authors were able to reveal systemic phenotypic impacts resulting from antigen re-stimulation in vitro, and the unimpacted ex vivo phenotype of antigen-reactive T cells.

A thorough analysis revealed that stable phenotypes correlate with various stimulation-induced major variations in transcriptomic signatures [107]. Details of the unimpacted ex vivo phenotypes of SARS-CoV-2-reactive T cells in peripheral blood were also revealed. Matching phenotypes between the peripheral blood antigen-reactive T cells and COVID-19 patients’ respiratory tracts were also reported. The study also revealed significant insights into the intercellular communication among respiratory T cells, antigen-reactive signatures, and macrophages that are positive for SARS-CoV-2 [107].

### 5.5. Host Lung and Colon Transcriptomes Show Elevated Neutrophil Extracellular Traps and TGF-β Response, Respectively

Transcriptomic analysis of COVID-19 patients by Wu et al. revealed several interesting observations as described below [108]. They identified 4065 DEGs in lung tissue, where 1470 genes were upregulated and 2595 were downregulated. Intriguingly, the analysis of lung tissue at the time of death of COVID-19 patients detected a scarce presence of SARS-CoV-2, which indicates the cause of death was likely sequelae that were associated with a host inflammatory response or COVID-19 pneumonia, and not severe active viral infection [108]. Gene ontology (GO) enrichment analysis of the transcriptomic results identified several genes such as myeloperoxidase, lactoferrin, and histones, which lead to the formation of neutrophil extracellular traps (NETs). The study detected the colocalization of myeloperoxidase, neutrophil elastase, and cytoplasmic DNA in foci, indicating the presence of NETs in deceased COVID-19 patients’ lungs [108]. It is believed that the observed NETs result from platelets, because the autopsies reveal an extremely high occurrence of thromboses, and this was not from SARS-CoV-2 infection, due to the presence of scarce viral transcripts. Indeed, a drastic elevation of platelet factor 4, which triggers NETosis, was observed [108].

The study also performed a transcriptomic analysis of colon tissue to understand the colon-related complications in COVID-19 patients. The analysis detected 4932 DEGs, where 1246 genes were upregulated and 3686 were downregulated. Interestingly, genes with an elevated expression correlated with their responses to transforming growth factor beta (TGF-β) indicating towards a local colon-specific response to TGF-β. Among the upregulated pathways of the colon and lung, only two groups were common e.g., extracellular structure organization and ossification [108]. Hence, SARS-CoV-2 targets of the colon transcriptome may likely have developmental significance.

### 5.6. Insights from Single-Cell Transcriptome of SARS-CoV-2 Infection in African Green Monkeys

African green monkeys were inoculated with a tissue culture infectious dose (TCID_50_) of replication-competent SARS-CoV-2 virus, and sgRNA from SARS-CoV-2 was detected in swab tests of inoculated animals. The observation indicates that viral replication likely took place in their respiratory tract [109]. Lung tissue from the inoculated animals indicated an early inflammatory response due to their subtle alveolar thickening, and the lower respiratory tract was identified as a site of SARS-CoV-2 replication from RNA detection studies [109]. Among lung cells, pneumocytes appeared to be the primary cells that were involved in productive virus replication. Transcriptomic perturbations due to infection were also detected in cells that were isolated from the lungs of infected animals, along with the observation that the mediastinal lymph nodes became inflammatory at 3 days post infection, showing enlargement [109]. At 10 days post infection, some of the mediastinal lymph nodes showed mild to moderate follicular hyperplasia, and all showed rare mononuclear cell immunoreactivity [109].

### 5.7. Lessons from the SARS-CoV-2 Transcriptome

The studies described in the above sections focused on the effect of SARS-CoV-2 infection on the host transcriptome and the significant information obtained from the specific transcriptional signatures of the host. In addition to the host transcriptome, mRNA transcripts of SARS-CoV-2 are also being investigated to expand our understanding of COVID-19. In this direction, Liu et al. analyzed single-cell transcriptomic profiles of moderate and severe COVID-19 patients’ samples and detected SARS-CoV-2 transcripts in multiple cell types as described below. SARS-CoV-2 transcripts were detected in lung epithelial cells and immune cells, such as macrophages, plasma cells, T cells, and NK cells, when the transcriptomes of BAL fluids from COVID-19 patients were analyzed [110]. The results indicated that all of the above cell types are susceptible to SARS-CoV-2 infection. The observations in the lung epithelial cells of severe COVID-19 patients show a high degree of inflammation and desquamation [110].

Single-cell transcriptomic analysis of severe versus moderate COVID-19 patients’ samples show a differential expression of four viral DEGs–N, ORF1ab, ORF3a, N, and ORF10, where only the severe samples showed the presence of ORF 10 [110]. In contrast, ORF10 was scarce in the BAL fluid of patients with moderate COVID-19. Hence, the observations indicated that ORF10 may serve as a potential biomarker for COVID-19 progression, due to its differential expression in severe versus moderate COVID-19 samples [110]. Analysis of single-cell and bulk transcriptomic datasets also revealed viral RNA fusion events. 

Even prior to COVID-19, transcriptional regulatory sequences associated with CoV gene expression had gained attention. One example is the coronaviral leader (TRS-L) sequence-dependent fusion transcripts of the classic sgRNA, which have a heptameric template-switching signal motif (TCTAAAC) [117]. A motif in the SARS-CoV-2 genome, which is one nucleotide longer (TCTAAACG), was found in the analysis of patients’ samples [110]. The authors also detected a high frequency of TRS-L independent fusions, and since some of the patients with these detected fusions passed away, certain fusions can likely predict worse outcome of patients with COVID-19 [110].

Apart from viral genes, the analysis also focused on host genes and detected 6 DEGs that are overexpressed in infected versus uninfected lung tissue transcriptome of patients with moderate COVID-19, namely—*BPIFA1, FCGBP, RARRES1, FAM3D, CD55*, and *CTSC* [110]. When the authors analyzed results from patients with severe COVID-19, only *BPIFA1* was overexpressed. Based on the known roles of *BPIF1A*, such as its antimicrobial activity, lung neutrophil infiltration, and interferon signaling for acute inflammation [110,118], its overexpression likely regulates an inflammatory response to SARS-CoV-2 infection in the lungs.

The remaining five DEGs also play roles in inflammation, immunity, and development. *FCGBP* encodes the Fc fragment of IgG binding protein, *RARRES1* encodes the retinoic acid receptor responder 1 to negatively regulate cell proliferation, *FAM3D* is associated with cytokine activity, *CD55* regulates complement cascade, and *CTSC* activates serine proteinases in cells of the immune system [110]. Among other tissues, the brain has been studied in COVID-19 research, where 65,309 single-cell transcriptomes from 30 frontal cortex and choroid plexus samples from 8 COVID-19 patients and controls revealed broad cellular disruptions in the direction of COVID-19-associated neurological pathologies [119]. Overall, the transcriptomic changes in cells that have been detected in the above studies indicate that cellular programming and development is also potentially affected by SARS-CoV-2 infection. In addition to the transcriptome, research has shown that SARS-CoV-2 infection also affects the epigenome, metabolome, and proteome [120,121,122,123,124,125,126,127,128,129,130,131,132,133,134,135].

## 6. RNA-Based Approach to Combat COVID-19

The impact of RNA-based research is not only confined to the understanding of COVID-19, but has also been significantly leveraged in the therapeutic domain in the development of vaccines against COVID-19. Technological advances have enabled RNA-based vaccines against COVID-19 to be developed at an unprecedented speed [136]. The process of RNA vaccine development involves sequencing of the viral RNA, followed by insertion of the sequence in a DNA template, containing flanking regulatory regions such that the corresponding RNA strands can be synthesized and packaged into lipid nanoparticles [10]. The RNA loaded into these vaccines can code for some of the viral proteins in the host cell, which elicit an immune response from the host such that antibodies against SARS-CoV-2 proteins are produced in the host which can combat a future viral infection.

A modified version of conventional RNA vaccines is where self-replicating vaccine candidates, containing replicase genes and instructions for the self-replication of RNA, are loaded onto vaccines [10]. Table 1 presents a few clinical trials from the United States against COVID-19 that have focused on RNA-based vaccines using BNT162b1/2/2SA [137,138,139] and mRNA-1273 [14,140,141], other types of vaccines which are based on virus vectors, e.g., Ad26.COV2.S [142,143,144], ChAdOx1 nCoV-19 [145,146], and whole-virion inactivated vaccines e.g., BBV152 [147,148]. Despite the success achieved by the above vaccines, areas of improvement include their stability outside of cold storage, immunogenicity and reactogenicity [149,150].

## 7. Conclusions

As we continue to expand our understanding of COVID-19 pathogenesis to develop effective therapeutics which currently do not exist, the knowledge regarding SARS-CoV-2 virology and its modes of cellular trafficking, such as exosomes, will provide potential information on how to effectively target SARS-CoV-2 propagation post-infection. An added layer of complexity is rendered by the evidence of in utero transmission of SARS-CoV-2 from infected pregnant mothers to fetuses, which is not well-understood.

The impacts of SARS-CoV-2 on the transcriptome provide a large amount of information which needs to be effectively utilized. The transcriptomic profiles reveal genes which are up/downregulated by the infection, such that they can be therapeutically targeted to reverse the effect. These profiles can also serve as biomarkers of the extent of infection, from mild to severe. These profiles further inform how cellular development and programming are impacted by SARS-CoV-2 infection. To address the knowledge gaps in understanding maternal to fetal transmission mechanisms, inheritance patterns of epigenetic imprints can be compared between cells of COVID-19-infected mothers and their progeny. It will be interesting to see if imprints of epigenetic perturbations in the mother induced by SARS-CoV-2 infection are inherited by offspring. Overall, the studies compiled in this review aim to expand our outlook towards diverse aspects of COVID-19, which can also benefit our understanding of other viral diseases, and help us better prepare against possible outbreaks in the future.

## Figures and Tables

**Table 1:** Clinical trials of RNA-based and other vaccines against COVID-19.

Clinical Trial Identifier	Intervention	Description
NCT04368728	BNT162b1	Lipid-nanoparticle-formulated, nucleoside-modified mRNA vaccine, encodes trimerized RBD of S protein of SARS-CoV-2
NCT04847050	mRNA-1273	Lipid nanoparticle–encapsulated mRNA-based vaccine, encodes prefusion-stabilized full length S protein of SARS-CoV-2
NCT04889209	Ad26.COV2.S	Recombinant, replication-incompetent human adenovirus type 26 vector encoding prefusion-stabilized full-length S protein of SARS-CoV-2
NCT04516746	ChAdOx1 nCoV-19 (AZD1222)	Replication-deficient simian adenovirus vector ChAdOx1 + full-length S protein SARS-CoV-2
NCT04834869	BBV152	Whole-virion inactivated, formulated with toll-like receptor 7/8 agonist adsorbed to alum

## Data Availability

Not applicable.
